# Current treatment options for leptospirosis: a mini-review

**DOI:** 10.3389/fmicb.2024.1403765

**Published:** 2024-04-25

**Authors:** Pavlo Petakh, Payam Behzadi, Valentyn Oksenych, Oleksandr Kamyshnyi

**Affiliations:** ^1^Department of Biochemistry and Pharmacology, Uzhhorod National University, Uzhhorod, Ukraine; ^2^Department of Microbiology, Virology, and Immunology, I. Horbachevsky Ternopil National Medical University, Ternopil, Ukraine; ^3^Department of Microbiology, Shahr-e-Qods Branch, Islamic Azad University, Tehran, Iran; ^4^Broegelmann Research Laboratory, Department of Clinical Science, University of Bergen, Bergen, Norway

**Keywords:** *Leptospira interrogans*, antibiotic, corticosteroid, probiotic, leptospirosis

## Abstract

Leptospirosis, one of the most common global zoonotic infections, significantly impacts global human health, infecting more than a million people and causing approximately 60,000 deaths annually. This mini-review explores effective treatment strategies for leptospirosis, considering its epidemiology, clinical manifestations, and current therapeutic approaches. Emphasis is placed on antibiotic therapy, including recommendations for mild and severe cases, as well as the role of probiotics in modulating the gut microbiota. Furthermore, novel treatment options, such as bacteriophages and newly synthesized/natural compounds, are discussed, and the findings are expected to provide insights into promising approaches for combating leptospirosis.

## 1 Introduction

Although leptospirosis is endemic in tropical geographic areas, it is known as one of the most common global zoonotic bacterial infections and may lead to large-scale epidemics resulting from flooding and strong rainfall (Haake and Levett, 2015; Rajapakse, 2022; Petakh et al., 2024a). The neglected zoonotic infection of leptospirosis is caused by *Leptospira* spp., a bacterial genus belonging to the gram-negative bacterial family *Leptospiraceae* within the bacterial Phylum *Spirochaetota* and the order *Leptospirales* (Cilia et al., 2021). According to the free-to-use service of the list of prokaryotic names with standing in nomenclature (Parte et al., 2020), the bacterial genus *Leptospira* encompasses 68 valid child taxa (species). The name Leptospira is rooted in the Greek words leptos (thin) and speira (helix).

The helical and coil-shaped *Leptospira* spp. are characterized by their typically folded pointed end, which seems to be a hook. The motile bacterial cells of *Leptospira* spp. are 6–20 μm in length and 0.1 μm in diameter. Indeed, they rotate through the presence of two periplasmic axial flagella, which are situated beneath the bacterial cell membrane (Cilia et al., 2021; Rajapakse, 2022; Fraga et al., 2024). Although Leptospira, the causative agent of leptospirosis, was first described by Stimson in 1907, leptospirosis infection was first described by Adolph Weil in 1886. In this regard, severe leptospirosis was named Weil’s disease. Weil syndrome manifests exclusively in severe cases of leptospirosis, characterized by renal dysfunction alongside hepatomegaly, liver dysfunction, and/or changes in consciousness levels (Jamal Khan et al., 2018; Petakh and Nykyforuk, 2022; Petakh et al., 2022a,b, 2023, 2024a). According to reports recorded in ancient texts, the other names for leptospirosis are rice field jaundice (in ancient China), autumn fever (Akiyami) in Japan and cane-cutter and swine-herd disease in Europe (Rajapakse, 2022).

The prevalence of leptospirosis in tropical regions, particularly East-Sub-Saharan Africa, Oceania, Southeast Asia and the Caribbean, is 73% (Costa et al., 2015; Rajapakse, 2022). Leptospirosis infection can occur and spread among people who have special jobs and occupations, e.g., military personnel, water sports participants and athletes, fishermen, rural farmers, slaughterhouse workers, veterinarians, and sewage workers, or among urban slum residents and vulnerable populations with low-level sanitation and poor housing (Parra Barrera et al., 2023; Philip and Ahmed, 2023). This situation increases human contact with leptospirosis in infected rats. As previous reports have shown, leptospirosis is the main bacterial agent of pulmonary hemorrhage syndrome (PHS) and is known as a global life-threatening infectious disease (Costa et al., 2015; Haake and Levett, 2015; Rajapakse, 2022; Parra Barrera et al., 2023). It may also lead to life-threatening acute kidney injury (AKI; Parra Barrera et al., 2023). Despite the presence of a wide range of reservoir hosts, including domestic and wild animals, *Rattus norvegicus* (or the brown rat) is the main source of leptospirosis in human hosts. The infection can be transmitted via the reservoir via direct or indirect contact (Haake and Levett, 2015). An infected reservoir keeps *Leptospira* within its kidneys. According to this knowledge, Leptospira exits the host’s body through shedding within the urine and continues its life cycle within the environment. Then, it can be acquired by a new host body. Blood-borne *Leptospira* can be disseminated into the kidneys. In this process, the peritubular capillaries or glomeruli are involved. The presence of *Leptospira* within the renal tubular lumen of the kidney may lead to leptospiral colonization (Haake and Levett, 2015).

Each year, zoonotic leptospirosis infections may result in approximately 1 million cases, with a mortality rate of around 6.86%, leading to approximately 60,000 deaths worldwide, as reported by Costa et al. (2015), Bradley and Lockaby (2023), and Shirzad et al. (2023). In accordance with the results reported by Costa et al. (2015), the major group of human hosts both at infection and at death were adult men aged between 20 and 49 years. The highest rates of morbidity and mortality associated with leptospirosis were detected in geographical areas affected by global burden disease (GBD), e.g., the Andes, Caribbean, Central and Tropical Latin America, East Sub-Saharan Africa, Oceania, and South and Southeast Asia.

A survey performed by Parra Barrera et al. (2023) revealed that 85.6% of the patients with leptospirosis in Colombia between 2015 and 2020 were men, with a mean age of 36.7 years.

Another study by Gizamba et al. (2023) investigated the incidence and distribution of leptospirosis in western Cape Province, South Africa, between 2010 and 2019. The results showed that 68.1% of the patients were males, and the remaining 31.9% were females, with a mean age of 37.0 years. They concluded that there was a significant correlation between the incidence of leptospirosis in humans and the age and sex of the patients (Gizamba et al., 2023).

Considering that the transmission of leptospirosis depends from environmental factors, the risk of leptospirosis varies within a geographical region (Costa et al., 2015; Beri et al., 2021). In 50% of the suspected cases, laboratory testing and confirmation procedures are not performed; due to this knowledge, this feature can be recognized as a significant challenge regarding the dissemination of leptospirosis and a possible ineffective treatment in this regard (Costa et al., 2015). As mentioned above, leptospirosis is a climate-sensitive, poverty-dependent and environment-borne infection (Costa et al., 2015; Davignon et al., 2023).

Parra Barrera et al. showed that antibiotic therapy was not administered to all patients with leptospirosis. Ceftriaxone, doxycycline, ampicillin, and penicillin (50.0%, 14.9%, 7.4%, and 5.2%, respectively) were administered to most patients. Furthermore, patients who received antibiotic therapy for severe leptospirosis were not included (Parra Barrera et al., 2023). Antibiotic therapy for inpatients with leptospirosis may include intravenous ampicillin, cefotaxime, ceftriaxone and penicillin (Panaphut et al., 2003; Haake and Levett, 2015). On the other hand, adult outpatients are orally administered azithromycin or doxycycline in the early period of leptospirosis. Amoxicillin or azithromycin is orally consumed by both children and pregnant females (Hospenthal and Murray, 2003; Haake and Levett, 2015). According to previous studies, the administration of doxycycline reduces the duration of infection (by 2 days), improves the incidence of infection and prevents the shedding of *Leptospira* in patients’ urine (McClain et al., 1984; Haake and Levett, 2015).

Although *Leptospira* spp. are susceptible to a wide range of antimicrobial agents, such as fluoroquinolones, macrolides, ß-lactams, streptomycin and tetracyclines, there are limitations related to the long duration of incubation, difficulty in accurate growth quantification and the application of serum in bacterial culture media. Despite these problems, the use of microdilution methods has facilitated the prophylaxis and treatment of leptospirosis (Haake and Levett, 2015).

*Leptospira* spp. exhibit intrinsic resistance to various antimicrobial agents, though the specific mechanisms responsible remain unidentified (Adler et al., 1986; Vinod Kumar et al., 2016; Petakh et al., 2024a,b). Nevertheless, resistance to sulfonamides, neomycin, actidione, polymyxin, nalidixic acid, vancomycin, and rifampicin has facilitated the development of selective media for isolating leptospires (Schönberg, 1981). The apparent absence of significant antimicrobial resistance emergence in *Leptospira* prompts the question of why this has not occurred (Liegeon et al., 2018). Leptospiral infections are typically monomicrobial, limiting opportunities for horizontal resistance gene acquisition. Moreover, there is no experimental evidence of foreign DNA uptake by *Leptospira* spp., although genomic analyses support this notion. Finally, human leptospirosis is a dead-end infection, with human-to-human transmission being extremely rare (Trott et al., 2018).

## 2 Current treatment options

In most cases, leptospirosis is characterized by mild clinical signs, which can improve spontaneously (Haake and Levett, 2015; Chacko et al., 2021). The treatment procedures used for leptospirosis are directly related to the condition (severity) of the infection (Wang et al., 2007; Monahan et al., 2009; Lucheis and Ferreira, 2011; Klaasen et al., 2014; Grassmann et al., 2017a; Gopi et al., 2021). Normally, the administration of oral doxycycline is recommended for the treatment of mild leptospirosis. In this regard, it is recommended to consume a dose of 100 mg doxycycline twice a day for a week. Amoxicillin (500 mg/day for 1 week to 10 days), ampicillin (500–750 mg/day for 1 week to 10 days), and azithromycin (500 mg/day for 3 days) can also be orally administered (McClain et al., 1984; Hospenthal and Murray, 2003; Pappas and Cascio, 2006; Charan et al., 2013; Kumar, 2013; Karpagam and Ganesh, 2020; Chacko et al., 2021). This therapeutic procedure results in a shorter disease duration (Fraga et al., 2024). In addition to the therapeutic role of the consumption of doxycycline in patients with leptospirosis, this antibiotic can be administered to those who travel to geographical zones that are recognized as endemic areas for leptospirosis. Moreover, doxycycline should be used by individuals with certain occupations, e.g., water sports athletes and veterinarians. In this regard, individuals are administered oral doxycycline weekly at a dose of 200 mg. Antibiotic consumption should be continued during the risk of exposure. This antibiotic may reduce the severity of leptospirosis and does not have a prophylactic role in the prevention of leptospirosis (Takafuji et al., 1984; Gonsalez et al., 1998; Sehgal et al., 2000).

**Figure 1:**
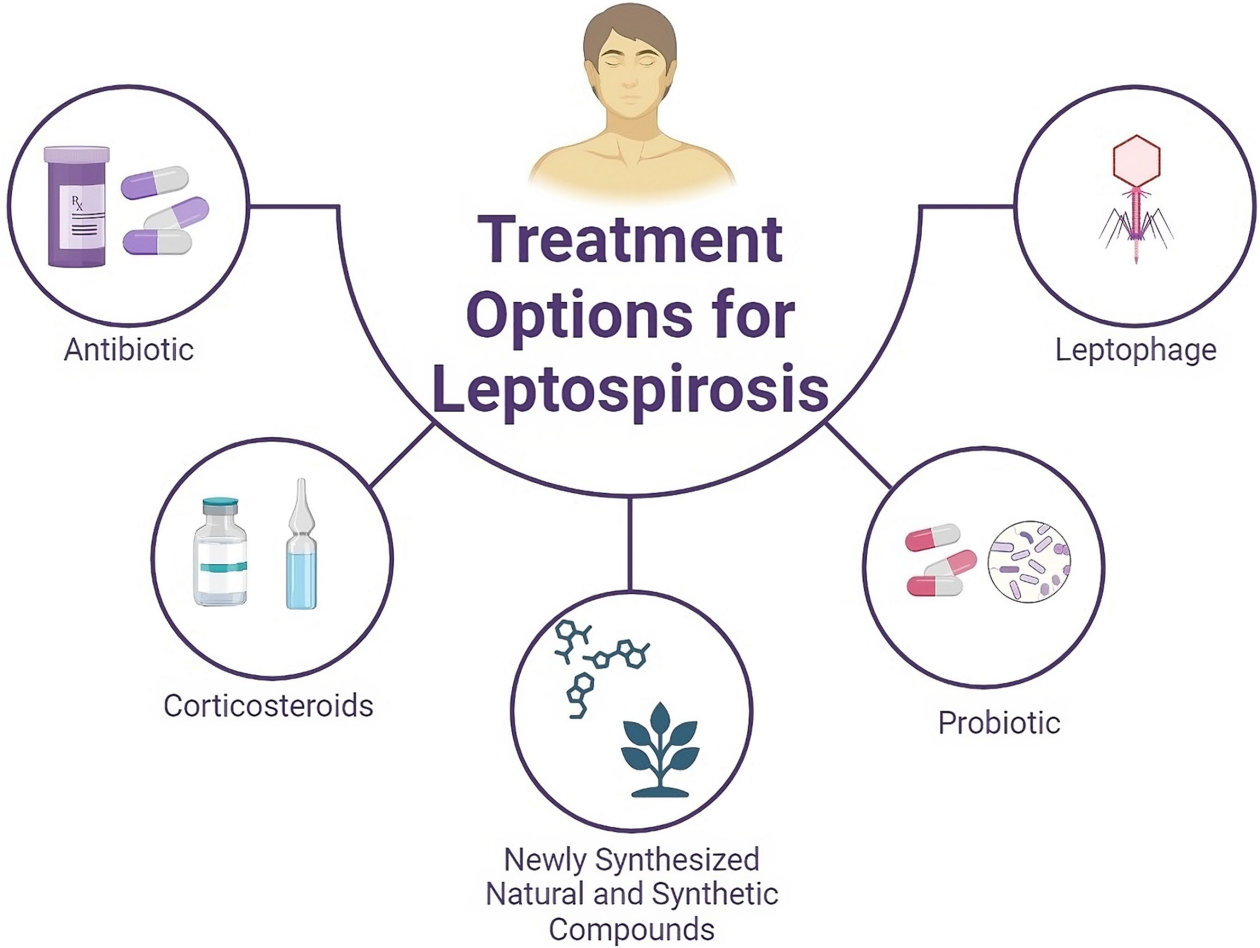
Schematic overview of current and potential therapeutic options for treating leptospirosis. In addition to antibiotics and steroids, natural compounds derived from medicinal plants, synthetic compounds, and probiotics can be considered for the treatment of leptospirosis.

Antibiotic therapy may be a good choice for the treatment of leptospirosis. Patients with severe leptospirosis, which normally manifests as renal and hepatic failure, are administered penicillin G (penicillin G sodium; at a dose of 1.5 million U/6 h) intravenously (Figure 1). It should be used for a week (Watt et al., 1988; Griffith et al., 2006; Karpagam and Ganesh, 2020; Guzmán Pérez et al., 2021). In accordance with reported records, amoxicillin, ampicillin, azithromycin, doxycycline and tetracycline can also be used for the treatment of severe leptospirosis. It is recommended that children and pregnant women avoid the consumption of doxycycline (Griffith et al., 2006; Jamal Khan et al., 2018; Gopi et al., 2021; Guzmán Pérez et al., 2021). Amoxicillin and azithromycin should be administered to children and pregnant women instead of doxycycline (Haake and Levett, 2015; Karpagam and Ganesh, 2020; Chacko et al., 2021).

To maintain homeostatic electrolyte and fluid conditions, supportive therapy is recommended. In this regard, hypomagnesemia has been observed in patients with severe leptospirosis (Spichler et al., 2008; Craig et al., 2009). Patients with leptospirosis who have severe pulmonary manifestations should be checked carefully because the mortality rate is high. Therefore, by observing pulmonary hemorrhage in patients with leptospirosis, antimicrobial therapy should be administered in parallel with respiratory ventilation as a mechanical therapy (Fraga et al., 2024).

In addition to the aforementioned antibiotics, the antibiotics cefotaxime and/or ceftriaxone are effective choices for the treatment of leptospirosis (Panaphut et al., 2003; Suputtamongkol et al., 2004). Despite the high efficacy of antibiotic therapy in association with leptospirosis, in some cases, Jarisch-Herxheimer reactions (JHRs) may be detected in some patients. JHR is a transient immunological phenomenon commonly seen in patients during treatment for leptospirosis, syphilis, and other spirochete infections. It manifests clinically with short-term constitutional symptoms such as fever, chills, headache, and myalgias. The appearance of JHR was observed 24 h after the consumption of antibiotics. This feature can be identified as a global concern in the field of antibiotic therapy for the treatment of leptospirosis (Friedland and Warrell, 1991; Dhakal and Sbar, 2022).

Although leptospirosis can be treated with a wide range of antibiotics, some antibiotics are not suitable for treating leptospirosis. Due to this knowledge, *Leptospira* spp. are not sensitive to chloramphenicol, metronidazole, rifampicin or vancomycin (Faine et al., 1999; Morgan, 2004).

Control and prevention are important measures that can be considered effective options for reducing the spread of leptospirosis. Promotion of hygiene and reduction of environmental contamination through the control of rodents in both zones of rural and urban areas are effective options for controlling the spread of leptospirosis and the transmission of bacterial agents of *Leptospira*. Simultaneously, the use of vaccines to vaccinate animals (livestock and domestic) and individuals with risky occupations is an influential preventive method in opposition to leptospirosis (Hotez and Ferris, 2006; Reis et al., 2008).

Currently, the process of vaccine production for human vaccination is undergoing significant progress. Some countries, including Japan, Cuba, France and China, have tested human vaccines. These vaccines have been licensed for use in related countries (Sánchez et al., 2002; Yan et al., 2003; Martínez et al., 2004; Rodriguez-Gonzalez et al., 2004; González et al., 2006; Laurichesse et al., 2007; Yanagihara et al., 2007).

Bacterins, which are human and veterinary vaccines, are constructed by killing bacterial cells of *Leptospira* via formalin or heat. Furthermore, some Chinese vaccines are prepared from the leptospiral outer membrane (OM; Yan et al., 2003). Hence, leptospiral components, including OM proteins (OMPs) and lipopolysaccharides (LPS), have been identified as suitable candidates for producing vaccines. Because of the high diversity among leptospiral strains, providing a single universal vaccine cannot be feasible (Gamberini et al., 2005; Raja and Natarajaseenivasan, 2015; Grassmann et al., 2017b; Jorge et al., 2018; Karpagam and Ganesh, 2020). In addition, subunit vaccines are provided by Lig proteins, which are produced during host infection. Lig proteins contribute to biotic bacterial adhesion and bacterial escape from the host immune system (Choy et al., 2007; Castiblanco-Valencia et al., 2012; da Cunha et al., 2019; Techawiwattanaboon et al., 2019).

The development of a highly efficient vaccine for leptospirosis continues to be a challenge (Williams and Gobbi, 2002). The pathogen has developed strategies to avoid the defensive role of the complement system, replicate in the bloodstream, attach to host cells, and infiltrate organs and tissues more rapidly (Wang et al., 2007). The rapid colonization of multiple organs by the pathogen poses a significant risk to the host, necessitating the development of a safe and effective leptospirosis vaccine. Inanimate vaccines, which primarily provide protection through the immune response triggered by LPS on their surface, typically offer temporary protection against the specific serovars contained in the vaccine formulation (Koizumi and Watanabe, 2005). On the other hand, live-attenuated vaccines are capable of activating both the cellular and humoral immune responses, thereby aiding in the establishment of long-lasting immunity (Bashiru and Bahaman, 2018). Although the process of attenuation can have a negative impact on the antigenicity of live vaccines, it becomes more difficult when multiple serovars are targeted.

## 3 Role of probiotics in the treatment of leptospirosis

The human gastrointestinal (GI) tract harbors a highly diverse and intricate microbial community consisting of more than 10^14^ species that interact with the host and contribute significantly to various physiological functions, particularly in supporting health and development (Honda and Littman, 2016). Imbalances in this gut microbiome are implicated in numerous diseases, including metabolic, noncommunicable, and infectious diseases (Noce et al., 2019; Ancona et al., 2023). The gut microbiome is well recognized for its pivotal role in initiating, modulating, and regulating immune responses (Belkaid and Hand, 2014; Fujimura et al., 2016). It produces short-chain fatty acids (SCFAs) with anti-inflammatory properties, aiding in processes such as cell apoptosis, inhibition of tumor cell growth, and maintenance of mucosal barriers (Li et al., 2018). Given the high concentration of immune cells in the intestine, the gut microbiota significantly influences immune responses not only in the gut but also in other organs (Tripathi et al., 2018). Emerging research indicates a crucial interplay between the gut and organs such as the liver, kidneys, and lungs, which are often implicated in leptospirosis (Bingula et al., 2017; Stavropoulou et al., 2021; Petakh et al., 2022a). Although the mechanisms underlying this crosstalk remain largely unknown, investigations into the role of the gut microbiota in leptospirosis infection have revealed significant alterations in microbial composition, particularly an increased *Firmicutes*/*Bacteroidetes* ratio, following infection (Xie et al., 2022). Depletion of the gut microbiota with antibiotics exacerbated the *Leptospira* infection burden in organs, while fecal microbiota transplantation had the opposite effect (Xie et al., 2022).

Importantly, antibiotic treatment, while targeting infection, can lead to dysbiosis in the gut microbiota, characterized by reduced diversity, altered abundance of specific taxa (some potentially harmful bacteria that become dominant, e.g., *Clostridium perfringens*, *Staphylococcus aureus*, or *Clostridioides difficile*), changes in gene expression and metabolites, compromised resistance to harmful bacteria, and the emergence of antibiotic-resistant microbes (Neuman et al., 2018; Ramirez et al., 2020; Strati et al., 2021). Consequently, antibiotic-induced alterations in the gut microbiota disrupt host–microbe interactions, increasing susceptibility to acute gut infections (Strati et al., 2021; Duan et al., 2022).

Various studies have explored the potential of probiotics in mitigating *Leptospira interrogans* pathogenesis, offering insights into their immunomodulatory effects. In a murine model, pretreatment with live *Lactobacillus plantarum* demonstrated promising outcomes. Notably, repeated oral administration of *L. plantarum* restored normal body weight in infected mice, mitigated histopathological signs of disease, and modulated the inflammatory response (Potula et al., 2017). Analysis revealed alterations in immune cell profiles, including increased B-cell and CD4+ helper T-cell populations, along with shifts toward effector CD4+ helper T cells postinfection. Moreover, pretreatment augmented populations of monocytes and macrophages in lymphoid tissues, potentially orchestrating a complex response involving myeloid and T-cell subsets. Immunohistochemistry revealed enrichment of neutrophils and macrophages in kidney sections from pretreated infected mice, consistent with reduced leucocyte and T-cell infiltration, suggesting a possible association between these cellular responses and reduced pathogenesis.

Another study investigated the immunomodulatory effects of *Saccharomyces boulardii*, a probiotic known for enhancing anti-inflammatory cytokine production and immune cell activation (Silveira et al., 2017). In combination with DNA vaccines encoding leptospiral protein fragments, *S. boulardii* significantly increased antibody titres and upregulated IL-10 expression, particularly with pTARGET/ligBrep vaccination. These findings suggest a potential role for *S. boulardii* in enhancing humoral immune responses associated with DNA vaccination, offering a novel strategy to improve vaccine efficacy.

Furthermore, *Bacillus subtilis* strains have been shown to demonstrate antagonistic effects on various *Leptospira* serogroups (Neustroev et al., 2015). These strains induced lysis of multiple *Leptospira* strains through the production of bacteriocins and enzymes, offering potential applications in veterinary medicine and environmental disinfection to combat leptospirosis.

Probiotic bacteria and dietary supplements can potentially prevent or reverse antibiotic-associated gut microbiota dysbiosis (Kesavelu and Jog, 2023). However, a comprehensive systematic review and meta-analysis conducted by Hungarian scientists found that the results of their analysis do not support probiotic supplementation during antibiotic therapy to prevent low-diversity dysbiosis (Éliás et al., 2023). The meta-analysis of Shannon, Chao1, and observed OTUs diversity indices did not demonstrate a significant effect of probiotics on maintaining diversity (Éliás et al., 2023).

## 4 Phage for leptospirosis treatment

Recently, there has been a growing interest in bacteriophages as potential substitutes for antibiotics and their impact on bacterial evolution. However, there is still limited understanding regarding the diversity of phages within the *Leptospira* genus (Doss et al., 2017; Schiettekatte et al., 2018). To date, only a few phages have been identified, isolated, and characterized within this genus, including vB_LbiM_LE1 (LE1), vB_LbiM_LE3 (LE3), and vB_LbiM_LE4 (LE4; Girons et al., 1990; Kropinski et al., 2009). Investigations into prophages closely linked with LE4 in *Leptospira* genomes led to the detection of a corresponding plasmid in *L. interrogans* and a prophage-like region in the preliminary genome of a clinical strain of *L. mayottensis*. The utilization of long-read whole-genome sequencing unveiled the presence of a circular plasmid reminiscent of the LE4 phage within the genome of *L. mayottensis* (Zhu et al., 2015).

Girons et al. (1990) first isolated bacteriophages from *Leptospira* species in 1990, but their exploration remains limited. Schiettekatte et al. (2018) demonstrated that leptophages utilize lipopolysaccharides (LPS) as receptors on bacterial cells.

Due to their tendency to target specific hosts, ranging from infecting a limited number of bacterial strains to occasionally affecting multiple closely related bacterial genera, phages generally have minimal impact on beneficial bacteria that protect health (Hyman and Abedon, 2010). In contrast, many chemical antibiotics, with their broader range of effectiveness, often lead to complications like antibiotic-associated *Clostridium difficile* colitis or *Candida albicans* yeast infections (Skurnik et al., 2007). However, now we have not found clinical data about the usage of phages for treating leptospirosis.

Considering the presence of leptophages, it is likely that leptospires should have appropriate natural protection systems against phages to limit phage infection (Bernheim and Sorek, 2020). Guohui Xiao et al. described the presence of the CRISPR–Cas system; however, as is known, microorganisms have a whole arsenal of defense systems against phages (Xiao et al., 2019; Yuan et al., 2023). Antiphage defense systems exhibit a nonrandom distribution in microbial genomes, often forming “defense islands” where multiple systems cluster together (Makarova et al., 2013; Doron et al., 2018; Hochhauser et al., 2023).

## 5 Corticosteroids in severe leptospirosis

Leptospirosis is a potentially severe zoonotic disease that unfolds in two distinct phases (Xavier et al., 2022). The initial phase involves acute febrile bacteraemia, followed by a period of apparent improvement. However, the subsequent “immune” phase is marked by renewed fever and the emergence of complications, with 5%–15% of patients progressing to Weil’s disease, often presenting with pulmonary involvement, including Acute Respiratory Distress Syndrome (ARDS; Vieira and Brauner, 2002; Dolhnikoff et al., 2007; Gulati and Gulati, 2012).

Two systematic reviews were identified, one of which included a meta-analysis (Rodrigo et al., 2014; Duggal et al., 2015). The initial review by Rodrigo et al. (2014) presented a qualitative synthesis from four studies. However, the lack of detail regarding the search process raises concerns about potential omissions of important studies, impacting the reliability of their findings. Additionally, the absence of clear inclusion criteria for study type, participants, intervention, and outcome measures, coupled with the limited number and poor quality of studies, further undermines the validity of their conclusions.

Both systematic reviews encountered challenges regarding the consistency of corticosteroid treatment regimens and the definition of pulmonary involvement. Discrepancies in dosages, such as the high dose of dexamethasone used in one study compared to methylprednisolone in another, may have influenced outcomes, including higher rates of nosocomial infections. Furthermore, variations in treatment frequency, duration, and concomitant medications add complexity to the interpretation of results.

Despite providing limited evidence and recommendations, Rodrigo et al. (2014) suggested potential benefits of early methylprednisolone administration for severe leptospirosis patients with pulmonary complications. However, this contrasts with the findings of Duggal et al. (2015), who reported no statistically significant differences in therapeutic effects among early high-dose corticosteroids (*p* ≥ 0.05; 95% CI: 0.81–1.37), early low-dose corticosteroids (*p* ≥ 0.05; 95% CI: 0.3–1.03), and late low-dose corticosteroids (*p* ≥ 0.05; 95% CI: 0.11–2.52). Although Duggal et al. presented a more comprehensive synthesis of randomized controlled trials, limitations such as the small number of studies and high statistical heterogeneity need to be addressed for further improvement.

In summary, the evidence regarding the efficacy of high-dose corticosteroids in severe leptospirosis patients with pulmonary complications remains limited. Additional well-designed randomized controlled trials with sufficient sample sizes are warranted. Attention to methodological aspects, including diagnostic criteria, disease severity definition, treatment uniformity, and outcome measurement consistency, is essential for future studies to provide robust evidence on this topic.

## 6 Newly synthesized/natural compounds of spirocidal agents

In recent years, there has been a surge in research focused on identifying novel synthetic and natural compounds with spirocidal activity against *Leptospira* species. These efforts encompass both preclinical and clinical studies aiming to explore potential treatment options for leptospirosis, a potentially life-threatening zoonotic disease. Herein, we provide an overview of the findings from various studies investigating the efficacy of different compounds against *Leptospira*, shedding light on promising avenues for future therapeutic interventions.

### 6.1 Synthetic compounds

Moon et al. (2007) conducted a study investigating the effectiveness of a short course of doxycycline and azithromycin in a lethal hamster model of leptospirosis. Their findings demonstrated a significant survival benefit with azithromycin treatment, particularly when administered once daily for 5 days. Similarly, Gopi et al. (2017) synthesized new oxime Schiff base derivatives and observed remarkable spirocidal activity against *Leptospira intrerrogans* serovar Icterohaemorrhagiae, highlighting the potential of these compounds as therapeutic agents. Additionally, Natarajan et al. evaluated novel azetidinones bearing quinoxaline derivatives and reported high inhibitory activity against *Leptospira*, suggesting their candidacy for further investigation (Selvaraj et al., 2013).

Ilangovan et al. (2017) described the synthesis of new pyrano derivatives and their efficacy against *Leptospira interrogans* serovar Autumnalis, indicating promising spirocidal activity. Furthermore, Ramalakshmishmi et al. synthesized 4-aryl 3-chloro N-pyridine 2-yl 2-azetidinones and found them to exhibit significant inhibition against *Leptospira intrerrogans* serovar Icterohaemorrhagiae, underscoring their potential as therapeutic candidates (Puratchikody, 2009).

### 6.2 Natural compounds

Several studies have explored the spirocidal activity of natural compounds derived from medicinal plants. Arulmozhi et al. investigated the ethanolic extract of *Andrographis paniculata* (commonly known as creat or green chiretta) leaves and found it to possess spirocidal activity against various *Leptospira* species, suggesting its potential as an alternative treatment option (Natarajaseenivasan, 2017). Similarly, Ishak et al. (2019) reported the spirocidal activity of extracts from *Canarium odontophyllum* (locally known as dabai in Sarawak and kembayau in Sabah and Brunei) leaves against *Leptospira*, highlighting the therapeutic potential of natural remedies.

In a recent publication, Nagarajan et al. (2014) conducted an investigation on the chloroform extract of *Piper betle* leaves (from the Piperaceae family). They discovered that this extract exhibited spirocidal activity when tested using the minimum inhibitory concentration (MIC) and microdilution method, with observations made using darkfield microscopy. The extract demonstrated substantial efficacy against various serovars of *Leptospira interrogans*, with concentrations ranging from 17.5 to 500 μg/mL. The MLC value of the extract is twice as high as the MICs. This extract is contraindicated for individuals with a history of liver disorder and dysfunction. A comparison was made between the activity of the chloroform extract from the leaves of *Piper betle* and a standard drug.

Prabhu et al. (2008) evaluated the spirocidal activity of *Eclipta alba* (commonly known as false daisy, yerba de tago, guntagalagara aaku, Karisalankanni, and bhringraj) extracts using tube dilution and microdilution techniques, demonstrating excellent efficacy against multiple *Leptospira* species. Additionally, Umamaheswari et al. (2010) investigated the spirocidal activity of *Seenthil sarkarai* and *Nilavembu kudineer* extracts, revealing significant inhibitory effects against *Leptospira*, further supporting the potential of natural compounds as therapeutic agents.

## 7 Conclusion

Effective treatment strategies for leptospirosis are multifaceted, ranging from antibiotic therapy to emerging options such as probiotics and novel compounds. Antibiotics such as doxycycline remain the cornerstone of treatment, with recommendations tailored to the severity of infection. Probiotics show promise in modulating the gut microbiota and augmenting immune responses, although further research is needed to delineate their precise role in leptospirosis management. Additionally, the exploration of bacteriophages and novel compounds presents exciting avenues for future therapeutic interventions. Continued efforts to enhance our understanding of leptospirosis pathogenesis and treatment modalities are crucial for mitigating the impact of this pervasive zoonotic disease on global health.

## Author contributions

PP: Conceptualization, Writing – original draft. PB: Writing – original draft. VO: Writing – review & editing. OK: Supervision, Writing – review & editing.
